# Schizophrenia hospitalization in the US 2005–2014: Examination of trends in demographics, length of stay, and cost

**DOI:** 10.1097/MD.0000000000025206

**Published:** 2021-04-16

**Authors:** Ethan Chen, Shahrzad Bazargan-Hejazi, Chizobam Ani, David Hindman, Deyu Pan, Gul Ebrahim, Anaheed Shirazi, Jim E. Banta

**Affiliations:** a Charles Drew University of Medicine and Science and David Geffen School of Medicine at University of California at Los Angeles (UCLA); b Department of Psychiatry; Charles Drew University of Medicine and Science & David Geffen School of Medicine at University of California at Los Angeles (UCLA); c Department of Internal Medicine, Charles Drew University of Medicine and. Science & University; d Department of Psychiatry; Charles Drew University of Medicine and Science; e Charles Drew University of Medicine and Science; f Department of Psychiatry, University of California at San Diego; g Health Policy and Leadership, School of Public Health, Loma Linda University, Los Angeles CA.

**Keywords:** age disparity, hospitalization cost, racial disparity, schizophrenia disparity, schizophrenia hospitalizations, schizophrenia trends

## Abstract

Primarily we aimed to examine the crude and standardized schizophrenia hospitalization trend from 2005 to 2014. We hypothesized that there will be a statistically significant linear trend in hospitalization rates for schizophrenia from 2005 to 2014. Secondarily we also examined trends in hospitalization by race/ethnicity, age, gender, as well as trends in hospitalization Length of Stay (LOS) and inflation adjusted cost.

In this observational study, we used Nationwide Inpatient Sample data and International Classification of Diseases, Eleventh Revisions codes for Schizophrenia, which revealed 6,122,284 cases for this study. Outcomes included crude and standardized hospitalization rates, race/ethnicity, age, cost, and LOS. The analysis included descriptive statistics, indirect standardization, Rao-Scott Chi-Square test, *t*-test, and adjusted linear regression trend.

Hospitalizations were most prevalent for individuals ages 45–64 (38.8%), African Americans were overrepresented (25.8% of hospitalizations), and the gender distribution was nearly equivalent. Mean LOS was 9.08 days (95% confidence interval 8.71–9.45). Medicare was the primary payer for most hospitalizations (55.4%), with most of the costs ranging from $10,000-$49,999 (57.1%). The crude hospitalization rates ranged from 790–1142/100,000 admissions, while the US 2010 census standardized rates were 380–552/100,000 from 2005–2014. Linear regression trend analysis showed no significant difference in trend for race/ethnicity, age, nor gender (*P* > .001). The hospitalizations’ overall rates increased while LOS significantly decreased, while hospitalization costs and Charlson's co-morbidity index increased (*P* < .001).

From 2005–2014, the overall US hospitalization rates significantly increased. Over this period, observed disparities in hospitalizations for middle-aged and African Americans were unchanged, and LOS has gone down while costs have gone up. Further studies addressing the important disparities in race/ethnicity and age and reducing costs of acute hospitalization are needed.

## 1 Introduction

Schizophrenia is a chronic, relapsing, mental disorder with an estimated prevalence of ∼ 1% in the US.^[1,2]^ and is among the top 15 leading causes of disability in the world.^[3]^ In 2013, the economic burden of schizophrenia was estimated to be $155.7 billion in the US.^[4]^ Patients with schizophrenia have reduced life expectancy, increased disability^[5,6]^ an increased risk of dying prematurely due to chronic co-morbid medical conditions,^[7–9]^ and a high suicide rate of 4.9% compared to the general population.^[10]^

Evidence of racial disparities in receiving diagnosis of schizophrenia is evident in research.^[11]^ According to a report from a US national data non-Hispanic whites, and Hispanics were 3.15 times more likely to receive schizophrenic diagnosis.^[12]^. In a different study African American, in compared to their white counter parts were over three times more likely to be diagnosed with schizophrenia.,^[13]^ a finding that is supported in other studies.^[13–16]^

Age of onset and gender are accepted as having powerful influence on its clinical progression and prognosis.^[17–19]^ The average age of onset for schizophrenia is 18 in males and 25 and 35 in females.^[20]^ Early age of onset,^[21,22]^ and co-morbid condition, among other factors, partake in hospitalization for schizophrenia.^[23]^ They also are risk factors for having poorer prognosis with longer hospital stays, more frequent hospital admissions, and a higher likelihood of readmission upon discharge, and subsequently substantial economic burden for the patient.^[2,24,25]^ While there are no gender differences in the prevalence of schizophrenia, but there is gender difference in age of onset.^[26]^ Males are 1.4 times more likely to be diagnosed compared to females.^[27,28]^

In general, the trends in rates of acute hospitalization can give us a metric of how effectively the condition is being treated. From 1996 to 2007 in the US, Blader investigated the trend in acute psychiatric hospitalizations, including schizophrenia, and found increasing psychiatric hospitalizations for children, adolescents, and adults while there was a decline in such rate for the elderly. The investigators, however, did not utilize a nationally representative sample.^[29]^

Of the studies conducted outside of the US to investigate schizophrenia-related hospitalization trends, few have utilized a nationally representative sample.^[30–33]^ Subsequently, this study's primary aim is to examine the crude and standardized schizophrenia hospitalization rates in the US from 2005–2014. We hypothesized that there would be a statistically significant linear trend in hospitalization rates for schizophrenia from 2005–2014. Additionally, we also examined trends in hospitalization by race/ethnicity, age, gender, and trends in hospitalization Length of Stay (LOS) and inflation-adjusted cost. Secondly, we hypothesized a significant difference among age groups, race/ethnicity, gender, LOS, and hospital cost. Our findings could improve our current understanding of patterns of schizophrenia among populations within the US. This knowledge is needed to identify risk factors further and delineate strategies for adequate provision of patient care, reduce hospitalization, LOS, therefore, reduce the health care cost, or least rethink resources distribution.

## 2 Methods

### 2.1 Study design and data

We conducted a cross-sectional study design using the Nationwide Inpatient Sample (NIS) database, which is a large hospital-based administrative national dataset, including hospital discharges from 2005 to 2014. The NIS was developed as part of the Healthcare Cost and Utilization Project (HCUP), a federal-state-industry partnership sponsored by the Agency for Healthcare Research and Quality. The NIS was designed to approximate a stratified 20% sample of all nonfederal, short-term, general, and specialty hospitals serving adults in the United States. The sampling strategy selected hospitals within states that have state inpatient databases according to defined strata based on ownership, bed size, teaching status, urban/rural location, and region. All discharges from sampled hospitals for the calendar year were then selected for inclusion into NIS. To allow extrapolation for national estimates, both hospital and discharge weights are provided. Detailed information on the design of the NIS is available at http://www.hcup-us.ahrq.gov. From 2005 to 2014, NIS captured discharge-level information on diagnoses, procedures, vital discharge status, and demographics.

Participants with missing data on any of the study variables were excluded from the analysis. Data elements that could directly or indirectly identify individuals were excluded, and all hospitalizations and discharges were independent. Thus, the unit of analysis was the hospital discharge rather than the individual patient. The study was exempted from review by the university institutional review board.

### 2.2 Patient selection

To analyze schizophrenia hospitalizations, we identified and included all discharges for which the age of the patient hospitalized was 18 or older with International Classification of Diseases, Eleventh Revisions -Diagnostic and Statistical Manual of Mental Disorders, 4th Edition (DSM-IV) codes of schizophrenia as indicated in the 1st column in Table 1. We controlled for confounding variables by excluding hospitalizations with one day or less and psychosis induced by substance or alcohol use by excluding International Classification of Diseases, Eleventh Revisions DSM-IV codes listed in the exclusion column in Table 1.

**Table 1:** ICD-9-DSM-IV inclusion and exclusion codes.

Inclusion ICD-9 codes	Exclusion ICD-9 codes
295.0 - Simple type schizophrenia	292.2 - Pathological drug intoxication
295.1 - Disorganized type schizophrenia	292.1 - Drug-induced psychotic disorders
295.2 - Catatonic type schizophrenia	292.11 - Drug-induced psychotic disorder with delusions
295.3 - Paranoid type schizophrenia	292.12 - Drug-induced psychotic disorder with hallucinations
295.4 - Schizophreniform disorder	292.89 - Other specified drug-induced mental disorders
295.5 - Latent schizophrenia	292.9 - Unspecified drug-induced mental disorder
295.6 - Residual type schizophrenia	305.3 - Hallucinogen abuse
295.7 - Schizoaffective disorder	305.6 - Cocaine abuse
298.8 - Other specified types of schizophrenia	305.7 - Amphetamine or related acting sympathomimetic abuse
295.9 - Unspecified schizophrenia	305.9 - Other, mixed, or unspecified drug abuse
	291.0 - Alcohol withdrawal delirium (291.0)
	291.3 - Alcohol-induced psychotic disorder with hallucinations
	291.4 - Idiosyncratic alcohol intoxication
	291.5 - Alcohol-induced psychotic disorder with delusions
	291.9 - Unspecified alcohol-induced mental disorders
	291.89 - Other alcohol-induced mental disorders
	291.81 - Alcohol withdrawal, withdrawal syndrome or symptoms
	969.6 - Poisoning by psychodysleptics
	969.7 - Poisoning by psychostimulants
	969.8 - Poisoning by other specified psychotropic agents
	969.9 - Poisoning by unspecified psychotropic agent

Other variables used in the analysis were as follows:

(1) demographic variables including age, gender, race, and ethnicity;
(2) total in-hospital charges
(3) insurance status described as Medicare, Medicaid, private insurance, and other;
(4) length of hospital stay;
(5) in-hospital mortality and
(6) Charlson's comorbidity index (CCI), derived from the HCUP medical comorbidity classification system.

The CCI was developed in 1987 and is used to measure the burden of co-morbidity^[34]^ by assessing 19 different disease comorbidity categories, each allocated a weight of 1 to 6 based on the adjusted relative risk of 1-year mortality and summed to provide a total score that is an indicator of disease burden and a reliable estimator of mortality.^[35]^

### 2.3 Statistical analysis

We computed descriptive statistics for all variables. To investigate age-related trends in hospitalizations for schizophrenia, we constructed three age groups of 18 to 44, 45 to 64, and 65+ and standardized these age groups to the 2010 the US census age groups.

We performed bivariate analysis through the Rao-Scott Chi-square test and *T*-test to detect any significant differences in the primary variables over time. Subsequently, we conducted an linear regression trend analysis to explore any changes from 2005 to 2014 in hospitalizations for schizophrenia.

Appropriate analytical adjustments were made using sample stratum, cluster, and discharge weight variables consistent with design and analysis requirements for the NIS data sample poststratification to the US population. Standard errors (SE) for the computed statistics were reported using methods the NIS dataset analysis methods. All data analyses were conducted using SAS 9.1 (SAS Institute, Cary, NC). Statistical hypotheses were tested using α less than 0.01 as the threshold for statistical significance. Data are presented with weighted n ± SE, weighted % ± SE, or mean with 95% confidence interval.

## 3 Results

After incorporating the study inclusion and exclusion criteria, the total number of hospitalizations for schizophrenia were 1,483,791 and, post-extrapolation to obtain a nationwide estimate yielded a weighted sample of 6,122,284 (Table 2). Slightly over thirty eight percent (38.8%) of the hospitalization cases were in the 45–64 age group, 25.9% were African American, 55.4% covered by Medicare, 24.4% had co-morbidity with chronic pulmonary disease, and 21.7% with uncomplicated diabetes, and mean CCI was 1.2 (1.17–1.24). The mean LOS was 9.08 days (8.71–9.45), and 57.2% of total in-hospital charges cost $10,000-$49,999. Mortality for individuals while being hospitalized for schizophrenia was 1.4% from 2005–2014.

**Table 2:** Demographic and basic characteristics of study sample.

Variables	Weighted *n*	Weighted % (SE)	
Age (yr)			
18–44	1647036	32.51 (0.54)	
45–64	1966026	38.81 (0.23)	
>/=65 yr	1453278	28.69 (0.60)	
Race			
White	3000973	59.23 (1.12)	
African American	1311140	25.88 (0.77)	
Hispanic	470020	9.28 (0.83)	
Other^∗^	284208	5.61 (0.34)	
Gender			
Male	2580702	50.94 (0.32)	
Female	2485683	49.06 (0.32)	
Co-morbid medical conditions			
Cerebrovascular disease	250198	4.94 (0.13)	
Chronic pulmonary disease	1234119	24.36 (0.32)	
Congestive heart failure	478081	9.44 (0.21)	
Connective tissue disease	59259	1.17 (0.03)	
Dementia	240368	4.74 (0.1687)	
Diabetes with complications	110006	2.17 (0.05)	
Diabetes without complications	1099976	21.71 (0.21)	
Metastatic carcinoma	81172	1.60 (0.05)	
Mild liver disease	159860	3.16 (0.07)	
Moderate to severe liver disease	33872	0.67 (0.03)	
Paraplegia/hemiplegia	62018	1.22 (0.03)	
Peptic ulcer disease	58228	1.15 (0.06)	
Peripheral vascular disease	149670	2.95 (0.08)	
Renal disease	328530	6.48 (0.16)	
Primary payer			
Medicare	2808950	55.44 (0.59)	
Medicaid	1354011	26.73 (0.60)	
Private	498989	9.85 (0.29)	
Other^†^	404390	7.98 (0.35)	
Total in-hospital charge			
<$10,000	1481012	29.23 (0.69)	
$10,000–$49,999	2895462	57.15 (0.46)	
>$50,000	689866	13.62 (0.45)	
Mortality			
Alive	4991029	98.65 (0.04)	
Dead	68440	1.35 (0.04)	Mean (CI)
Length of stay (d) - LOS			9.08 (8.71 – 9.45)
Charlson's co-morbidity index - CCI			1.20 (1.17 – 1.24)

**Figure 1:**
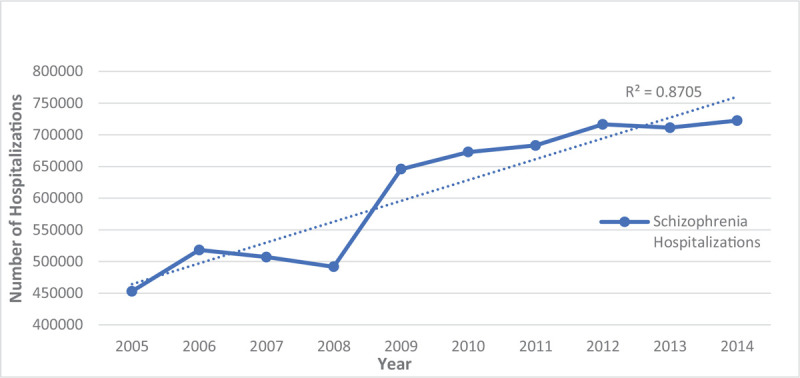
Hospitalizations for persons with schizophrenia from 2005–2014.

**Figure 2:**
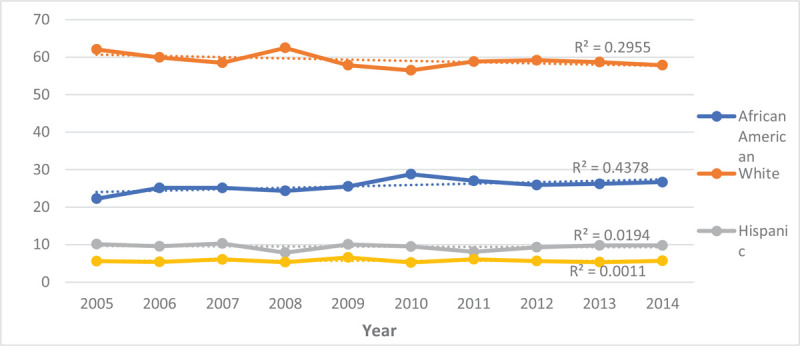
Percent hospitalizations for persons with schizophrenia by race (Crude) from 2005–2014.

**Figure 3:**
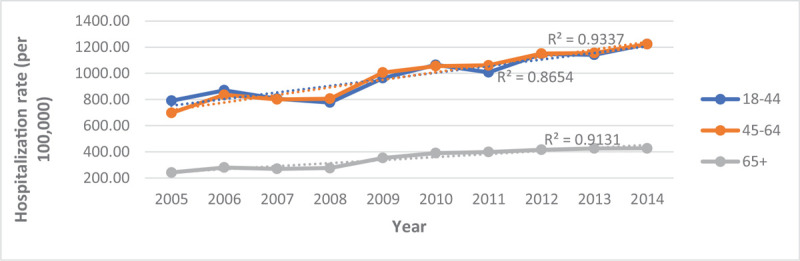
Crude hospitalization rate for persons with schizophrenia by age from 2005–2014.

**Figure 4:**
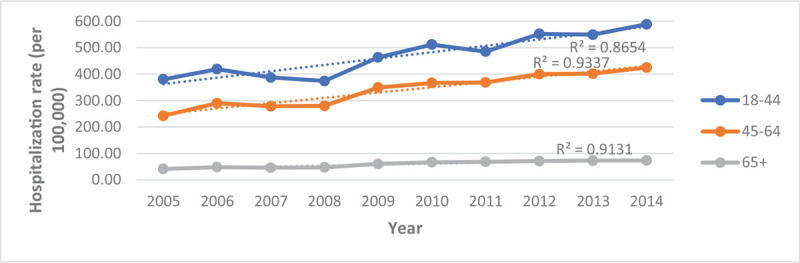
Age adjusted [post standardization] hospitalization rate for persons with Schizophrenia by Age from 2005–2014.

As indicated in Table 3 and Figure 1, the number of hospitalizations has been increasing from 2005 to 2014 from 453,020 to 722,415 hospitalizations, respectively. Despite the increasing hospitalizations, the percentage of hospitalizations by race/ethnicity were not significantly different throughout this decade (Fig. 2). When examining hospitalizations by age, we observed that the hospitalization rate for all age groups has increased before and after age standardization to the 2010 US Census age groups (Figs. 3 and 4). However, post standardization, there was an evident difference in the rate of hospitalizations between the 18 to 44 and 45 to 64 age groups (Fig. 4), whereas, before standardization, the rates were similar (Fig. 3).

**Table 3:** Schizophrenia patient characteristics by year (crude rates).

		2005	2006	2007	2008	2009	2010	2011	2012	2013	2014	Sig.
	Hospitalizations^∗^	453020	518333	507044	491863	645817	672878	683215	716530	711169	722415	
	18–44	34.56 (1.13)	33.08 (1.02)	32.56 (1.10)	30.76 (1.17)	30.67 (1.08)	30.83 (1.08)	29.43 (0.96)	30.69 (0.45)	30.96 (0.47)	31.28 (0.49)	
Age	45–64	37.30 (0.40)	38.96 (0.43)	39.51 (0.41)	39.36 (0.45)	40.62 (0.46)	40.28 (0.46)	40.18 (0.52)	40.36 (0.22)	40.07 (0.24)	39.91 (0.24)	*P*<.001
	>/=65	28.14 (1.20)	27.96 (1.14)	27.92 (1.25)	29.88 (1.31)	28.71 (1.21)	28.89 (1.23)	30.38 (1.12)	28.95 (0.50)	28.97 (0.49)	28.81 (0.49)	
	White	62.05 (2.32)	59.93 (1.82)	58.50 (2.42)	62.48 (2.22)	57.86 (2.03)	56.49 (2.30)	58.83 (1.67)	59.19 (0.80)	58.69 (0.82)	57.87 (0.83)	
	African American	22.26 (1.44)	25.14 (1.46)	25.12 (1.44)	24.34 (1.49)	25.52 (1.42)	28.79 (1.83)	27.02 (1.55)	25.90 (0.66)	26.19 (0.66)	26.68 (0.67)	
Race	Hispanic	10.11 (1.90)	9.55 (1.25)	10.31 (1.70)	7.88 (1.15)	10.07 (1.45)	9.49 (1.55)	8.06 (0.80)	9.28 (0.51)	9.80 (0.53)	9.79 (0.54)	*P*=.36
	Other^†^	5.58 (0.87)	5.38 (0.75)	6.07 (0.77)	5.31 (0.48)	6.55 (0.92)	5.23 (0.66)	6.09 (0.74)	5.63 (0.29)	5.32 (0.25)	5.67 (0.28)	
Gender	Male	49.90 (0.66)	50.95 (0.57)	51.11 (0.72)	50.06 (0.57)	52.09 (0.68)	52.20 (0.70)	51.40 (0.48)	52.19 (0.25)	52.56 (0.28)	52.88 (0.27)	*P*<.01
	Female	50.10 (0.66)	49.05 (0.57)	48.89 (0.72)	49.94 (0.57)	47.91 (0.68)	47.80 (0.70)	48.60 (0.48)	47.81 (0.25)	47.44 (0.28)	47.12 (0.27)	
LOS	(mean)	9.45 (0.38)	9.00 (0.27)	9.28 (0.47)	8.81 (0.27)	8.87 (0.40)	8.58 (0.39)	8.81 (0.31)	8.29 (0.11)	8.39 (0.11)	8.49 (0.11)	*P*=.06
	[95% CI]	[8.70–10.19]	[8.46–9.54]	[8.36–10.20]	[8.28–9.34]	[8.09–9.65]	[7.81–9.35]	[8.21–9.41]	[8.08–8.50]	[8.17–8.61]	[8.27–8.71]	
CCI	(mean)	1.07 (0.03)	1.15 (0.03)	1.17 (0.04)	1.23 (0.037)	1.30 (0.04)	1.30 (0.04)	1.40 (0.03)	1.37 (0.02)	1.37 (0.02)	1.40 (0.02)	*P*<.01
	[95% CI]	[1.00–1.13]	[1.09–1.20]	[1.10–1.24]	[1.16–1.30]	[1.24–1.37]	[1.22–1.38]	[1.34–1.47]	[1.34–1.40]	[1.34–1.40]	[1.37–1.44]	
Total in-hospital charge	$10,000	35.21 (1.48)	32.23 (1.35)	28.16 (1.21)	27.22 (1.40)	25.75 (1.40)	25.26 (1.48)	20.27 (1.17)	20.68 (0.57)	19.05 (0.57)	17.29 (0.55)	
	$10,000 - 49,999	54.44 (1.09)	57.13 (1.03)	58.37 (0.87)	59.34 (0.90)	59.50 (1.15)	58.79 (0.93)	60.94 (0.85)	60.96 (0.45)	60.75 (0.46)	61.45 (0.47)	*P*<.01
	$50,000	10.36 (0.73)	10.64 (0.63)	13.47 (0.94)	13.44 (0.84)	14.75 (1.07)	15.95 (0.99)	18.79 (0.97)	18.36 (0.42)	20.20 (0.46)	21.26 (0.48)	

**Figure 5:**
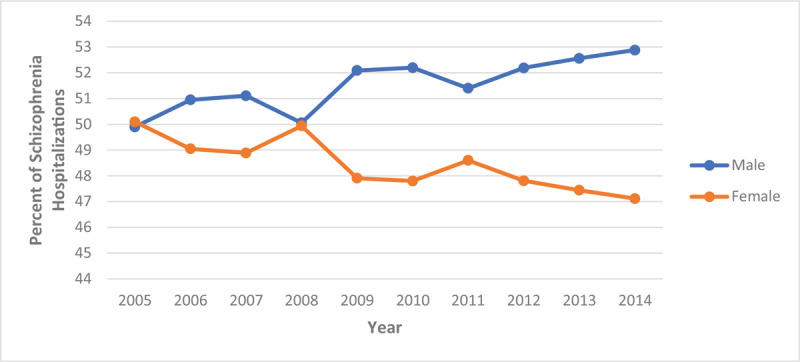
Schizophrenia hospitalization by gender (Crude) from 2005–2014.

**Figure 6:**
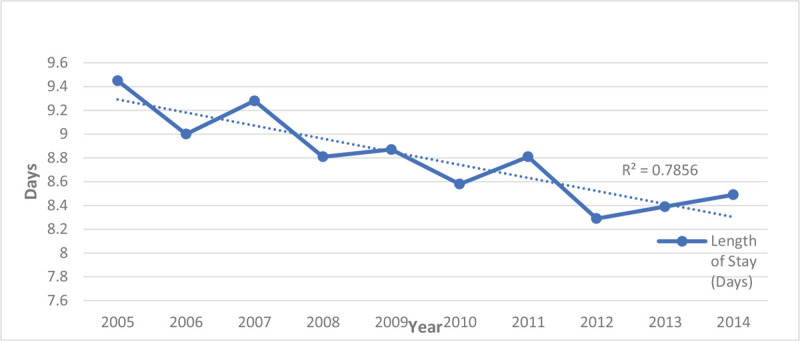
Mean length of stay of hospitalizations for patients with schizophrenia from 2005–2014.

**Figure 7:**
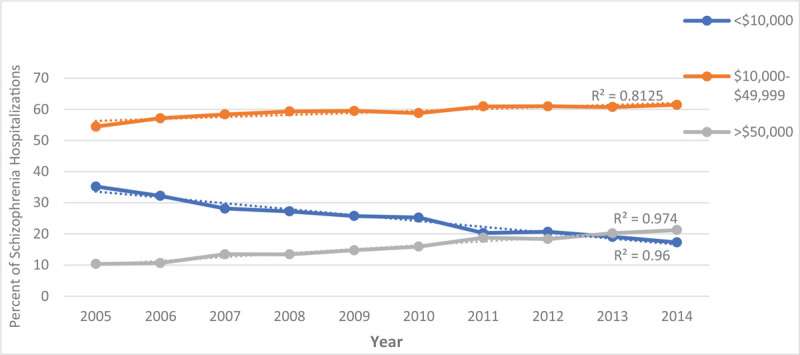
Total in-hospital charge for persons with schizophrenia from 2005–2014.

**Figure 8:**
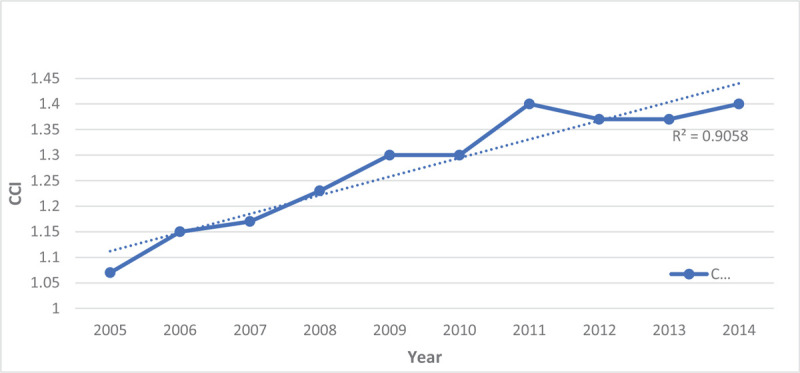
Mean Charlson's co-morbidity index of hospitalizations for patients with schizophrenia from 2005–2014.

From 2005 to 2014, the percentage of hospitalizations for males was up trending from 49.9% to 52.9% while, for females, it was down-trending from 50.1% to 47.4% (Table 3 and Fig. 5). Over this decade, the LOS for persons hospitalized with schizophrenia decreased by nearly a day (Fig. 6), and the percentage of charges were higher, with 61.5% of hospitalizations now costing between $10,000-$49,999 (Fig. 7). The percentage of hospitalizations costing ≥ $50,000 have more than doubled from 10.4% to 21.3% while hospitalizations costing < $10,000 declined from 35.2% to 17.3%. Charlson's Comorbidity Index has been slightly up-trending from 1.07 to 1.40 in this period (Table 3 and Fig. 8).

After performing linear regression, there was no significant difference among age groups, race/ethnicity, nor gender since P > 0.001 (Table 4). However, we found a significant difference when comparing age-adjusted hospitalization rates (*P* < .001). In addition, the difference for LOS, hospital cost and CCI was significant with *P* = .006, *P* < .001 and *P* < .001, respectively.

**Table 4:** Change in distribution and mean of study variables.

Variable	*P* value	Variable	*P* value
18–44 yr	.021	CCI	<.001
44–64 yr	.020	Male	.001
> 65yr	.177	Female	.001
White	.104	Hospitalization cost (Mean)	<.001
African American	.037	LOS	.001
Hispanic	.701	Age adjusted hospitalization rate/100,000	<.001
Other^∗^	.926		

## 4 Discussion

We found a statistically significant linear trend in hospitalization rates for schizophrenia from 2005–2014. This may in part, be due to increased access for patients with mental health disorders after approval of the Affordable Care Act since March 23, 2010.^[36]^ It may also be due to worsening of disease due to inadequate treatment, which is discussed by Chaudhari *et al.* as predominantly due to non-adherence with reasons that include medication side effects, polypharmacy, perception of treatment ineffectiveness, lack of finances, poor access to a nearby mental health facility, poor insight or poor social support.^[37]^ Recent changes in diagnostic criteria with the implementation of the DSM-V in 2013 can be disqualified in influencing our findings of increased hospitalizations since 99.5% of patients with schizophrenia diagnosed by the DSM-IV would also meet schizophrenia criteria in DSM-V.^[38]^

When looking at hospitalizations trend with respect to race/ethnicity, there was no real significant difference. However, African Americans continue to overrepresent with schizophrenia as evident in previous studies,^[15]^ encompassing 25% of hospitalizations due to schizophrenia, but only representing 13.4% of the US population.^[3]^ This continuous disparity in the burden of schizophrenia amongst African Americans should be addressed in future studies.

When examining hospitalizations trend with respect to age, we found that there was no significant change. However, when standardizing each age group to the 2010 US Census age groups, there was an increase in hospitalizations amongst all age groups, with the highest hospitalization rate for individuals 18 to 44 and lowest for individuals older than 65. Studies of Fitch et al and Huang et al, may explain this finding, which reported younger individuals constituting more inpatient visits compared to older individuals due to poor medication adherence.^[2,21]^ Early medication nonadherence was found to be a predictor of poor drug adherence that leads to the pattern of disease relapse and hospitalization that is sustained throughout an individual's life,^[39,40]^ which may explain the up-trending hospitalization rate for all standardized age groups. Furthermore, the decrease in hospitalization rate from younger to older age groups in every observed year could be due to low adherence in younger individuals. Young people underestimate the necessity of treatment, but as they age, they become more adherent as they acquire more insight about the illness course and necessity of treatment to remain symptom-free, resulting in fewer hospitalizations.^[37]^

Although there was no significant difference amongst gender in hospitalizations, we still saw an up-trend in hospitalization for males while there was a down-trend for females over this decade. This may be due to differences in the presentation of schizophrenia symptoms between males and females. Males are known to have more negative symptoms and more severe clinical features,^[26,28]^ therefore, higher indication of hospitalization. While females, approximately 50% fewer hospitalizations compared to men.^[41]^ Females have also shown to be more compliant with their medications, which leads to better treatment outcomes.^[42]^ Also, physiologic differences as a result of gender could be involved, where men require higher dosages of antipsychotics in contrast to women due to increased hepatic clearance.^[28,43]^

When looking at the mean LOS over this decade, there was a significant change. There was a decrease of nearly one day from 9.45 to 8.49 days. Comorbid substance use for patients with schizophrenia has previously been associated with a shorter inpatient stay, postulated to be due to amplification of schizophrenia symptoms by substance abuse and quick resolution of psychotic symptoms upon removal of the abused substance.^[44,45]^ However, we still saw a shorter LOS despite excluding hospitalizations with a substance use disorder. Further studies are needed to explore what factors influence LOS in this population. The treatment team can use this information in aligning the care plan accordingly.

Our findings revealed that the total in-hospital charges increased for most hospitalizations for schizophrenia. This could be due to inflation or that younger individuals constitute more inpatient visits compared to older individuals due to poor medication adherence leading to higher inpatient costs, as supported by others.^[2,21]^ The age-standardized hospitalization rates in our study of individuals 18 to 44 were the highest each year compared to the groups with older individuals and had been up-trending from 2005 to 2014, which is consistent with the higher proportion of younger individuals requiring more inpatient utilization. However, this conflicts with our finding of decreased LOS since nonadherence has been linked to slower recovery and longer LOS.^[45]^

Patients with schizophrenia have a higher risk of premature mortality from chronic co-morbid conditions.^[7]^ We observed a significant increase in mean CCI for individuals with schizophrenia from 2005–2014, with most co-morbidities being uncomplicated diabetes and chronic pulmonary disease. This could be attributed to a higher rate of smoking and heavy nicotine dependence in patients with schizophrenia.^[46]^ Additionally, long-term use of some of the anti-psychotic medications increases the risk of metabolic syndrome in schizophrenia patients.^[47]^ Overall, we found that the risk of in-hospital mortality was very low over this decade.

### 4.1 Limitations

Our study has several limitations due to the nature of cross-sectional study design and relying on prior coding of diagnosis through an existing administrative database. However, the NIS has been repeatedly studied as a national dataset from “real-world” situations. We cannot relate our findings to any process of care, given the limited scope of clinical data available in the NIS database, as the unit of analysis is the hospitalization and not the patient. We also are unable to determine if the primary reason for hospitalization is due to schizophrenia based on the diagnosis order list in NIS, nor are we able to distinguish initial hospitalizations from repeat hospitalizations. Moreover, we followed the Census age groups, but that may not reflect the true nature of hospitalization trends for schizophrenia. Nevertheless, we proceeded with our grouping to allow for comparability with other studies. Similarly, our groupings by dollar amount for the total in-hospital charges may also not reflect the true nature of the cost trends reflecting hospitalizations for schizophrenia.

## 5 Conclusions

We found a significant increase in the number of hospitalizations from 2005 to 2014. We also found that African Americans continue to be overrepresented with schizophrenia. Additionally, hospitalizations amongst all age groups increased with the highest hospitalization rate for the younger population and lowest for the older. We found no gender difference in hospitalization but an uptrend for males and a downtrend for females across the study period. Furthermore, the overall costs for schizophrenia-related hospitalizations increased while the LOS decreased in hospitalization cases. Based on these findings, further studies are needed to shed some light on strategies to achieve equity in the management of schizophrenia in different populations to reduce hospitalizations and its continuous substantial economic burden. Additionally, future studies should closely investigate key determinants of LOS to ensure payment system reflects the needs of different populations burdened by schizophrenia.

## Author contributions

**Conceptualization:** Shahrzad Bazargan-Hejazi, Chizobam Ani, David Hindman, Deyu Pan, Gul Ebrahim, Jim. E. Banta.

**Data curation:** Chizobam Ani.

**Formal analysis:** Chizobam Ani, Deyu Pan.

**Methodology:** Shahrzad Bazargan-Hejazi, Deyu Pan, Jim. E. Banta.

**Project administration:** Shahrzad Bazargan-Hejazi.

**Resources:** Jim. E. Banta.

**Supervision:** Shahrzad Bazargan-Hejazi, Chizobam Ani, David Hindman, Jim. E. Banta.

**Writing – original draft:** Ethan Chen, Shahrzad Bazargan-Hejazi, Chizobam Ani.

**Writing – review & editing:** Shahrzad Bazargan-Hejazi, Chizobam Ani, Anaheed Shirazi, Gul Ebrahim.
